# Minimally Invasive Treatment of Displaced Proximal Humeral Fractures in Patients Older Than 70 Years Using the Humerusblock

**DOI:** 10.1155/2016/6451849

**Published:** 2016-11-17

**Authors:** Robert Bogner, Reinhold Ortmaier, Philipp Moroder, Stefanie Karpik, Christof Wutte, Stefan Lederer, Alexander Auffarth, Herbert Resch

**Affiliations:** ^1^Department of Traumatology and Sports Injuries, Paracelsus Medical University, Müllner Hauptstraße 48, 5020 Salzburg, Austria; ^2^Paracelsus Medical University, Strubergasse 21, 5020 Salzburg, Austria; ^3^Center for Musculoskeletal Surgery, Charité-Universitaetsmedizin Berlin, Campus Virchow, 13353 Berlin, Germany; ^4^KH Oberndorf, Department of General Surgery, Paracelsusstraße 37, 5110 Oberndorf, Austria

## Abstract

*Background.* Surgical treatment of proximal humeral fractures (PHF) in osteoporotic bone of elderly patients is challenging. The aim of this retrospective study was to evaluate the clinical and radiological outcome after percutaneous reduction and internal fixation of osteoporotic PHF in geriatric patients using the semirigid Humerusblock device.* Methods.* In the study period from 2005 to 2010, 129 patients older than 70 years were enrolled in the study. After a mean follow-up of 23 months, a physical examination, using the Constant-Murley score and the VAS pain scale, was performed. Furthermore radiographs were taken to detect signs of malunion, nonunion, and avascular necrosis.* Results.* The recorded Constant-Murley score was 67.7 points (87.7% of the noninjured arm) for two-part fractures, 67.9 points (90.8%) for three-part fractures, and 43.0 points (56.7%) for four-part fractures. In ten shoulders (7.8%) loss of reduction and in four shoulders (3.1%) nonunion were the reason for revision surgery. Avascular humeral head necrosis developed in eight patients (6.2%).* Conclusions.* In two- and three-part fractures postoperative results are promising. Sufficient ability for the activities of daily living was achieved. In four-part fractures the functional results were less satisfying regarding function and pain with a high postoperative complication rate. In those patients other treatment strategies should be considered.* Study design.* Therapeutic retrospective case series (evidence-based medicine (EBM) level IV).

## 1. Introduction

Proximal humeral fractures (PHF) are common in elderly patients, with a six times higher incidence in patients above 70 years compared to younger patients [1]. Usually, treatment is often more difficult, than in a younger age group, due to preexisting osteopenia, osteoarthritis, and rotator-cuff lesions along with various systemic comorbidities [2, 3]. Therefore treatment of PHF in elderly patients is a challenge for the treating physicians.

In nondisplaced and minimally displaced fractures conservative treatment seems to be the preferable therapy rendering a good functional outcome [4]. In complex PHF results after conservative treatment in advanced aged patients are significantly worse. Therefore, surgical treatment is recommended [4, 5]. However, insufficient reduction of displaced fractures can lead to nonunion or malunion of the fragments leading to poor functional results.

Even though there are many studies, reporting on the outcome after surgically treated PHF in elderly patients, no clear treatment guidelines exist. Some studies favour osteosynthesis [6, 7] and some prosthetic replacement [8–10].

In this retrospective single centric study the technical feasibility as well as clinical and radiological outcome after minimally invasive, percutaneous reduction and internal fixation (CRIF) of displaced PHF in the elderly using the semirigid Humerusblock device is analyzed.

## 2. Patients and Methods

In the study period from January 2005 to December 2010, 261 PHF in patients above 70 years were surgically treated at an urban level I trauma center. The decision for surgical instead of conservative treatment was made according to modified Neer II criteria [11] and comprised displacement of the tuberosities greater than 5 mm or misalignment of the head fragment greater than 30 degrees.

232 fractures were treated using the minimally invasive Humerusblock device (Synthes, Oberdorf, Switzerland). Cannulated screws (Stryker Leibinger Micro Implants, Freiburg, Germany) were used to fix the tuberosity fragments. In 16 cases an intramedullary nail was implanted. A reversed shoulder arthroplasty (RSA) was performed in 12 shoulders (6 head split fractures, 4 fracture dislocations, and 2 four-part fractures). Although in our department we do not use routinely angular stable plates for treating PHF, in 1 patient open reduction and internal fixation with an angular stable plate was performed. In that patient the procedure was demonstrated as life operation during a convention by an experienced shoulder surgeon, who was not part of our medical staff.

In this study all patients above 70 years at the time of injury, who were treated with the Humerusblock device for PHF, were included in the study. Patients with injury-unrelated musculoskeletal impairment of the involved shoulder, neurologic diseases impairing shoulder function, and comorbidities preventing a clinical and radiological follow-up were excluded from this study.

Out of the 232 patients treated with the Humerusblock device, 207 patients were included. The mean age was 79.8 years (range, 70–101). The injury mechanism was simple fall in 191 patients (92.3%), skiing accident in nine patients (4.3%), bicycle accident in five patients (2.4%), and traffic accident in two patients (1%).

43 patients (20.8%) died and were lost to follow-up. Six patients (2.9%) came from abroad and were not available for clinical and radiological examination; 29 patients (14%) could not be reached for follow-up.

129 patients (62.3%) could be evaluated clinically and radiographically after a mean of 22.9 months (range, 6–79) postoperatively. There were 105 women (81.4%) and 24 men (18.6%). Ten of these patients were evaluated separately and included in the complication analysis as they had undergone revision surgery using a different technique.

The surgical treatment was performed 2.2 days after trauma on average (range, 0–19 days).

Radiological follow-up was completed at 2, 4, and 6 weeks postoperatively and at final follow-up. Radiographs were taken in anteroposterior (AP) and axial path of rays to evaluate signs of malunion, nonunion, and avascular necrosis (AVN).

The clinical outcome was evaluated using the Constant-Murley score [12]. The pain was assessed using a visual analogue scale (VAS). The ability to complete activities of daily living was rated on a 20-point scale. The range of motion (maximum 40 points) was evaluated using a goniometer in 5° increments. A force meter (IsoForceControl, MDS Medical Device Solutions AG, Oberburg, Switzerland) was utilized to measure the power in 90° of abduction (maximum 25 points). The overall outcome was compared to the uninjured arm and related to age- and gender-matched normal values.

### 2.1. Operative Technique

**Figure 1:**
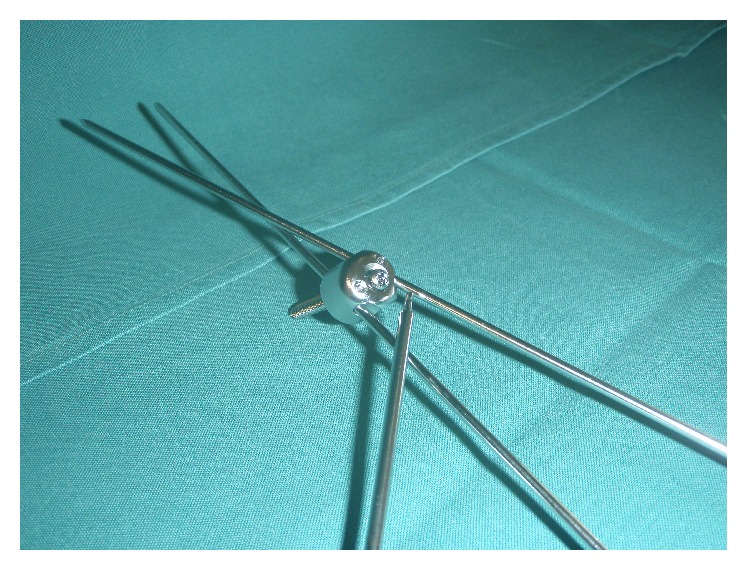
The Humerusblock device with the cannulated, self-tapping screw and two 2.2 mm k-wires.

**Figure 2:**
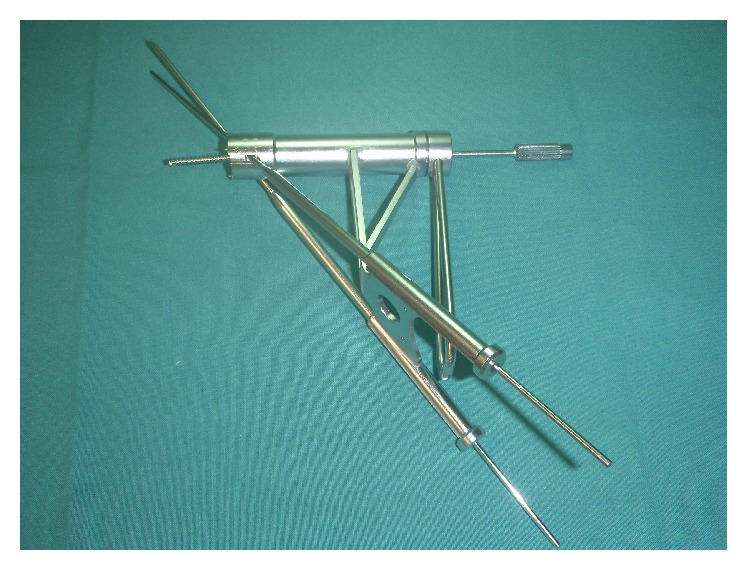
The aiming device. The k-wires are inserted percutaneously through the guiding sleeves.

**Figure 3:**
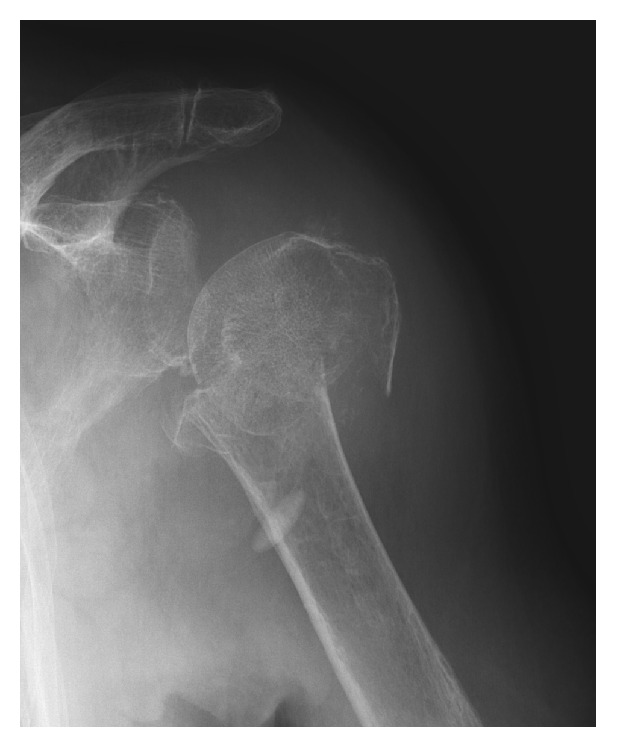
Radiograph of a 3-part proximal humeral fracture of a 96-year-old woman.

**Figure 4 fig4:**
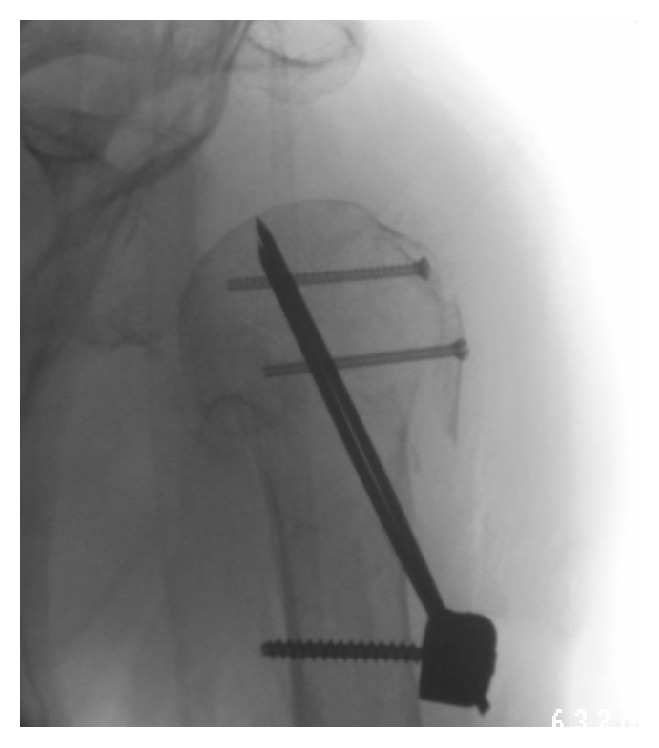
Intraoperative X-ray after reduction and stabilization using the Humerusblock.

**Figure 5:**
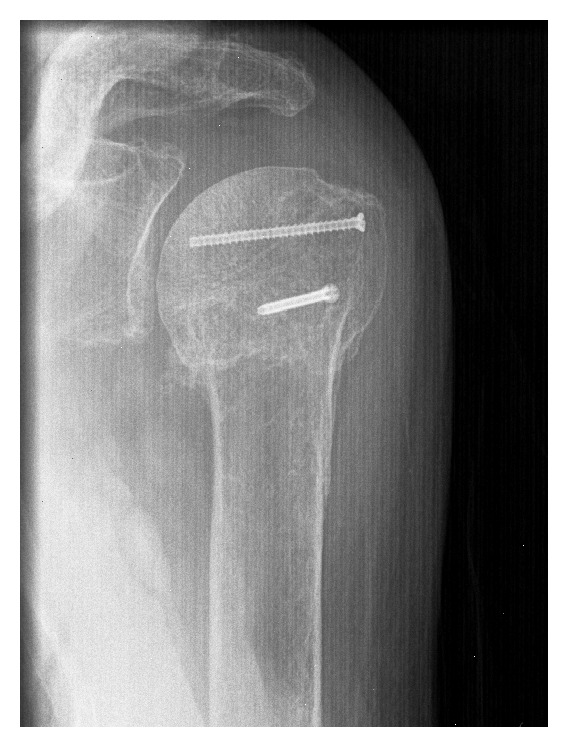
At time of follow-up 15 months after minimally invasive stabilization bony healing with a relative Constant-Murley score of 97% was observed.

The operative technique of CRIF for PHF using the Humerusblock has been widely described in the past [13–16]. The surgery is performed in beach-chair position and fluoroscopy is utilized for visualization of the reduction and fixation steps. A delta split approach of 3 cm is performed about 4 to 5 cm below the fracture site at the lateral aspect of the humerus. Then the Humerusblock is brought in and fixed to the shaft of the humerus by a 3.5 mm self-tapping, cannulated screw. It is very important to hold the arm in neutral rotation during introductions of the Humerusblock. Two k-wires are introduced to the humeral shaft through a guiding device (Figures 1 and 2). The two k-wires are brought in at an angle of 35° to the shaft and 25° to each other. This allows the k-wires to cross over each other and diverge in the humeral head. Before reduction the k-wires are drilled up to the subcapital fracture level. Then the fracture is reduced manually by traction and/or percutaneously by using an elevator via small stab incisions. In 3-part fractures the head fragment is derotated using a bone hook. After anatomical reduction the head fragment is fixed by introducing the two k-wires up to the subchondral bone. Finally the k-wires are locked within the Humerusblock. Additionally, displaced tuberosities are reduced with small bone hooks, introduced via stab incisions. After reduction the tuberosities are fixed with 3.0 mm cannulated screws (Figures 3 [Fig fig4]–5).

### 2.2. Postoperative Treatment

Postoperatively the upper arm is immobilized in a standard shoulder sling for three to four weeks. Beginning from the first postop day passive mobilization is recommended. Active movement is started after the fourth postoperative week. The removal of the Humerusblock is advised following bony healing after approximately six weeks.

## 3. Results

In patients with two-part fractures a mean postoperative Constant-Murley score of 67.7 points (range, 25–90) was recorded which accounted for 87.7% of the contralateral side. Compared with age- and gender-matched normal values, this equated to 100.6% of the anticipated score.

In patients with three-part fractures the mean postoperative Constant-Murley score was 67.9 points (range, 36–90), accounting for 90.8% of the noninjured arm, which results in 99.5% of the age- and gender-correlated score. In patients with four-part fractures the mean Constant-Murley score was 43.0 points (range, 18–62), which represents 56.7% compared to the noninjured side. The age- and gender-correlated Constant-Murley score was 63.7%.

The mean pain score for two-part fractures was 13.6 points (range, 5–15), for three-part fractures 13.5 points (range, 8–15), and for four-part fractures 12.2 points (range, 5–15).

The average achievable range of motion is illustrated in Table 2.

The mean operating time was 51.3 ± 29.5 min in the investigated patients. The mean total hospital stay was eight days (range, 3–20 days). The mean intraoperative fluoroscopy time was 183.4 ± 93.3 seconds.

Types of fracture of the included patients according to the AO/OTA and Neer classification are shown in Table 1.

### 3.1. Complications and Revisions

100 (77.5%) of all fractures treated with the Humerusblock showed primary bony union without complications. Secondary dislocation was recorded in ten patients (7.8%). In four of those patients, revision surgery, using the Humerusblock again was performed. The patients were included in the clinical follow-up. In four patients RSA, in one patient intramedullary nail, and in one patient an angular stable plate was used for revision surgery. Nine (7%) nonunions were observed; in four of those patients surgical revision was performed (three RSA, one blade-plate). One superficial, postoperative wound-infection was treated conservatively with intravenous antibiotics. In one patient deep vein thrombosis of the operated arm was observed. AVN of the humeral head occurred in eight shoulders (6.2%). Five (3.9%) showed partial necrosis and three (2.3%) showed complete collapse of the humeral head.

Table 3 shows an overview of all complications and corresponding therapies.

## 4. Discussion

In advanced age above 60 years, especially in women, bone density of the humeral head decreases significantly [2]. In PHF, the head fragment is usually very fragile with small amounts of subchondral bone, which often gives the impression of an “egg shell” [17]. Treatment of fractures in patients with weak bone is challenging. The aim of this study was to evaluate the functional and radiological outcome of surgical treatment of osteoporotic PHF in elderly patients, using a minimally invasive, semirigid device.

Surgical treatment strategies for severely displaced PHF comprise joint preserving methods, such as plate osteosynthesis, intramedullary nailing, k-wire based methods, and anatomical and reverse arthroplasty (RSA).

Hemiarthroplasty (HA) has long been the gold-standard in treating severely displaced PHF [10, 18, 19]. However, outcome after HA is strongly dependent on tuberosity healing [20, 21]. High rates of tuberosity resorption and bad functional outcome after HA for primary fractures treatment are reported [22, 23]. Due to its biomechanical features, functional outcome after RSA is more independent from tuberosity healing. Therefore, RSA gained popularity in fracture treatment of severely displaced PHF in elderly patients [22]. In a recently published systemic review, the authors found significantly better results for RSA than for HA in the treatment of PHF. However, complication rate was not significantly different [22, 24].

In a recent matched-pair analysis comparing RSA and CRIF using the Humerusblock for the treatment of three- and four-part fractures significantly better results were found in the CRIF group [16].

Alternative strategies to treat PHF are ORIF using plates as well as intramedullary nailing. Often, fixed-angled locking-plates are used, giving a sufficient primary stability in young patients for early mobilization of the shoulder. In osteoporotic bone secondary perforation or cut-out of the screws is reported in up to 43% [25–27]. This can lead to secondary loss of reduction and damage to the glenoid surface. Another risk of rigid implants such as fixed-angled locking-plates is the lack of flexibility not allowing sufficient sintering of the fragments. This potentially increases the secondary displacement rate and decreases the rate of bony union [28]. In osteoporotic bone, flexible implants, which reduce the forces on the implant-bone interface, show advantages in biomechanical studies [29, 30].

A fundamental principle of the Humerusblock is dynamic stabilization. So a guided sintering of the fragments along the k-wires to assure a continuous contact of the fracture fragments can be achieved. Consequently humeral head perforation of the k-wires is not unusual. In these cases, in order not to jeopardise the glenoid surface, shortening of the k-wires is necessary before starting active movements. In our patients, retrieval of perforating k-wires was necessary in 21.8%. Of course this leads to another operation and even though this can be done in a local anesthesia it still shows a disadvantage of the procedure.

In general a higher rate of AVN is described in literature after ORIF, mainly due to compromising soft tissue and the blood supply of the humeral head during surgery [31]. In closed reduction soft tissue bridges can be preserved. Periosteal bridges between shaft and head fragment especially are not disturbed through this technique. They often present the only left perfusion for the humeral head. This is validated in our data where we found a humeral head necrosis rate of 6.2% compared to 10% in open reduction techniques of a collective with an average age of 62 years [32]. 75% of all AVN were found in the group of four-part fractures. This suggests the consideration of primary arthroplasty in those special cases.

In the presented study all tuberosities healed. This is a better prerequisite if conversion to secondary arthroplasty will be necessary after occurrence of AVN. With anatomically healed tuberosities and an intact rotator cuff, anatomical shoulder replacement can be considered.

A revision rate of 10.9% in patients with the average age of 80 is compared to other implants in the lower range [32]. Taking a look at four-part fractures the reoperation rate increases to 35.7%. A conversion to RSA due to secondary displacement was necessary in four patients. In one case nonunion was treated with a blade-plate. The clinical results of four-part fractures treated with the Humerusblock are far below those of two- and three-part fractures.

A disadvantage in all semirigid implants is the necessity of immobilizing the shoulder. In younger patients an immobilization for about three weeks is adequate; in advanced age immobilization needs to be extended to four weeks. However, passive mobilization is started immediately after surgery.

Some limiting factors of the present study have to be mentioned. The main limitation is its retrospective design. Furthermore, a follow-up rate of only 62% could be achieved. However, all patients were of advanced age and most of them have comorbidities, which can explain the high dropout rate. Another limitation is the missing comparison group to other implants like angle-stable plates or intramedullary nails. A multicentre study is projected to compare different options of operative treatment of PHF in elderly patients.

## 5. Conclusion

Minimally invasive fracture treatment can be demanding especially in a poor bony situation. Of course this procedure has a certain learning curve. For complex PHF an experienced surgeon is needed to achieve the best possible outcome. Recognising the “personality” of the fracture before entering the operation is crucial to be able to identify the fragments and its periosteal bridges [33]. Subsequently the course of action is planned. To make planning easier we recommend a CT scan with 3-dimensional reconstructions preoperatively.

Analyzing the results of this study pain-free shoulder mobility with good range of motion can be achieved in a great number of patients after minimal invasive PHF treatment using the Humerusblock. In highly unstable four-part fractures a sufficient stabilization can be hardly achieved. Due to the high rate of AVN and the risk of secondary dislocation a RSA should be considered in this patient group.

## Figures and Tables

**Table 1:** Fracture division according to modified Neer's criteria and AO/OTA classification.

	Number of patients	Percent of total
Neer classification		
Two-part	63	52.9%
Three-part	47	39.5%
Four-part	9	7.6%
AO/OTA classification		
A-type	63	52.9%
B-type	39	32.8%
C-type	17	14.3%

**Table 2:** Values of range of motion and corresponding points according to the constant score.

Direction (medium, range)	Two-part (*n* = 63)	Three-part (*n* = 47)	Four-part (*n* = 9)
Flexion (°)	143 (45–180)	139 (80–180)	81 (40–115)
Abduction (°)	138 (50–175)	131 (50–175)	78 (40–115)
Internal rotation (points)	6.2 (2–10)	6.1 (0–10)	4.0 (0–6)
External rotation (points)	7.8 (0–10)	7.4 (0–10)	4.0 (0–8)

**Table 3:** Complication overview and therapy.

Complication	*N*	%	Therapy
Dislocation	10	7.8%	Reosteosynthesis with HB: 4
			RSA: 4
			Blade-plate: 1
			Intramedullary nail: 1
Nonunion	9	7.0%	RSA: 3
			Blade-plate: 1
			No further surgery: 5
AVN	8	6.2%	No further surgery: 8
Infection	1	0.8%	Conservative
Arm thrombosis	1	0.8%	Conservative

## Abbreviations

PHF: Proximal humeral fractures
CRIF: Closed reduction and internal fixation
RSA: Reversed shoulder arthroplasty
AP: Anteroposterior
AVN: Avascular necrosis
VAS: Visual analogue scale
HA: Hemiarthroplasty.
